# Prenatal Organophosphate Pesticide Exposure and Targeted Maternal Pregnancy Metabolomic Profiles in the NYU CHES Cohort

**DOI:** 10.1021/acs.est.5c05412

**Published:** 2025-10-10

**Authors:** Haleigh Cavalier, Akhgar Ghassabian, Sara E. Long, Yelena Afanasyeva, Susan Sumner, Susan McRitchie, Rachel Coble, Yu Chen, Kurunthachalam Kannan, Zhongmin Li, Mengling Liu, Leonardo Trasande

**Affiliations:** † Department of Population Health, 12297 NYU Langone Medical Center, New York, New York 10016, United States; ‡ Department of Pediatrics, Division of Environmental Pediatrics, NYU Langone Medical Center, New York, New York 10016, United States; § Department of Nutrition, UNC Chapel Hill, Chapel Hill, North Carolina 27516, United States; ∥ UNC Chapel Hill Nutrition Research Institute, Kannapolis, North Carolina 28081, United States; ⊥ 1094 Wadsworth Center, New York State Department of Health, Albany, New York 12237, United States; # NYU Wagner School of Public Service, New York, New York 10003, United States

**Keywords:** metabolomics, pregnancy, organophosphate, pesticide

## Abstract

Prior research links prenatal exposure to organophosphate (OP) pesticides to adverse health outcomes via molecular mechanisms, such as oxidative stress, neurotransmitter disruption, and mitochondrial dysfunction. This study investigates such mechanisms by assessing the relationships between prenatal OP pesticide exposure and targeted urinary maternal metabolomic profiles using data from the New York University Children’s Health and Environment Study (NYU CHES) cohort (*n* = 890). Urine samples were collected at three time points during pregnancy (*T*_1_, *T*_2_, *T*_3_) and analyzed for six dialkyl phosphate (DAP) metabolites and a targeted set of 188 metabolites related to key biological functions. Associations between DAP concentrations and individual metabolites were estimated using linear and logistic regression models, with adjustment for potential confounders. Statistical analysis revealed significant associations between OP exposure and the studied maternal metabolomic profile, with the most associations observed during late pregnancy (*T*_3_). The most robust associations across all models and time points were observed for acylcarnitine profiles, which were consistently altered in association with OP exposure. This study identifies specific metabolic signatures associated with OP pesticide exposure during pregnancy, providing insights into potential mechanisms of toxicity and implications for maternal and child health. Additional research is needed to validate these findings and further understand the functional significance of the identified metabolic disturbances.

## Graphical abstract

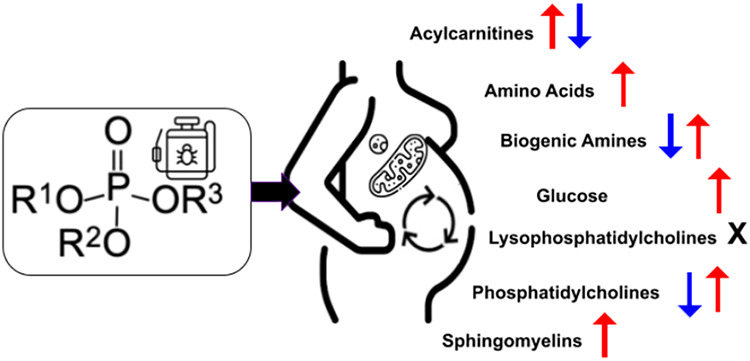

## Introduction

Over the past decade, global pesticide use has increased, and human exposure is widespread.^1–3^ Among these chemicals, organophosphate (OP) pesticides are some of the most extensively purchased and applied across diverse sectors.^4^ OPs are acutely toxic, primarily due to their capacity to inhibit acetylcholinesterase, a critical enzyme in nervous system function.^5–7^ In addition, through noncholinergic mechanisms, lower dose exposure has the potential for toxicity at levels relevant outside of the occupational setting, particularly when exposure occurs during a critical window of vulnerability, such as fetal development.^8–11^

Epidemiological and experimental studies conducted in animals and in vitro suggest associations between prenatal exposure to OPs and various neurodevelopmental health outcomes, such as cognitive/intellectual impairments and autism spectrum disorder (ASD),^9–19^ reproductive health outcomes such as semen quality and fetal growth restriction,^17,18,20^ and chronic diseases like cancer, and metabolic syndrome, among others.^17,21–23^

Oxidative stress, inflammation, mitochondrial dysfunction, neurotransmitter disruption, and altered energy metabolism can be triggered by OP pesticides and thus are proposed mechanisms for their impact on health outcomes.^24–26^ Though several pathways are suggested, the molecular mechanisms of chronic OP pesticide chemical toxicity remain unclear. Metabolomics is a tool, relatively new to molecular epidemiology, that produces rich, high-throughput data with the ability to identify metabolic signatures and disturbances associated with environmental exposures and intermediate markers of disease.^27,28^ The metabolome uniquely captures a physiological snapshot, including both endogenous and exogenous perturbations. With recent improvements in metabolite identification and functional analysis, metabolomics can be harnessed by environmental epidemiologists to discover exposomic biomarkers and probe proposed pathways to gain mechanistic insights.^27,29,30^

As such, leveraging data from a large pregnancy cohort, we utilized targeted metabolomics to examine associations between OP pesticides and metabolic pathway disturbances to elucidate their implications on health outcomes. We hypothesize that exposure to OP pesticides will be associated with disturbances in metabolic pathways in pregnant participants that are relevant to the aforementioned molecular mechanisms and health outcomes of the offspring.

## Materials and Methods

### Study Population

This research used data from the New York University Children’s Health and Environment Study (NYU CHES), an ongoing, prospective cohort study that enrolls pregnant people 18 years or older from NYU Langone Hospital-Manhattan, Bellevue Hospital, or NYU Langone Hospital-Brooklyn and follows them through the prenatal period. Biospecimens and questionnaire data are collected during prenatal care visits at three time points during pregnancy: early pregnancy (<18 gestational weeks, *T*_1,_), midpregnancy (18–25 gestational weeks, *T*_2_), and late pregnancy (>25 gestational weeks, *T*_3_). Of the 4439 participants currently enrolled in NYU CHES, 3577 provided urine samples during prenatal visits. Among participants who provided urine samples, 995 participants’ samples were analyzed for OP pesticide metabolites (*T*_1_ = 937, *T*_2_ = 718, *T*_3_ = 812), and 2230 participants’ samples were used for metabolomics analysis (*T*_1_ = 1947, *T*_2_ = 1465, *T*_3_ = 1757). Participants with at least one available measurement of OP pesticide and metabolomics at the same time point are included in this analysis (*n* = 890, *T*_1_ = 780, *T*_2_ = 691, *T*_3_ = 768). All included participants’ pregnancies occurred between 2016 and 2018. All participants enrolled in NYU CHES provided written informed consent. This study was approved by the Institutional Review Board of the New York University Grossman School of Medicine. The study design and cohort characteristics are described in further detail elsewhere.^31^ To assess potential selection bias, we compared characteristics of our study sample to the entire NYU CHES cohort and found no evidence of differential loss to follow-up (Supporting Information Table S1).

### Organophosphate Pesticide Analysis

Single-spot urine samples were collected at prenatal care visits (*T*_1_, *T*_2_, and *T*_3_) and aliquoted into polyethylene containers and stored at −80 °C until chemical analyses. Six dialkyl phosphate (DAP) metabolites were extracted from urine samples and characterized using HPLC-MS/MS. Urine extraction and metabolite quantification are described in detail elsewhere.^32–34^ Three dimethyl (DM) metabolites (dimethyl phosphate (DMP), dimethyl thiophosphate (DMTP), and dimethyl dithiophosphate (DMDTP)) were determined, as well as three diethyl (DE) metabolites (diethyl phosphate (DEP), diethyl thiophosphate (DETP), and diethyldithiophosphate (DEDTP)), though DEDTP was excluded from the main analysis, as it was only detected in 8% of samples. Mass sums of diethyl phosphates (∑DE), dimethyl phosphates (∑DM), and dialkyl phosphates (∑DAP) were calculated.^35^ The mass sum of all DAPs was used as a biomarker for total, nonspecific exposure to organophosphate pesticides, as most OP pesticides are metabolized into at least one DAP.^36^ Creatinine was measured in all samples, and DAPs were multiplied by the ratio of median creatinine in the group divided by the creatinine of the individual to adjust for urine dilution.^37,38^ The limits of detection (LOD) of DMP, DMTP, DEMTP, DEP, DETP, and DEDTP were 0.02, 0.005, 0.009, 0.075, 0.005, and 0.053 ng/mL, respectively. Values under the limit of detection (LOD) were replaced with the LOD divided by the square root of 2, a method supported when detection is high (>80%), as it is in our data.^39^

### Targeted Metabolomics Data

Targeted metabolomics was conducted on urine samples collected throughout pregnancy (*T*_1_, *T*_2_, and *T*_3_). Metabolites were identified and quantified via high-performance liquid chromatography (HPLC) and mass spectroscopy (MS) and a flow injection analysis (FIA) using the Biocrates AbsoluteIDQ p180 Urine Extension kit (Biocrates Life Sciences AG, Innsbruck, Austria). Absolute measurements for 188 metabolites (40 acylcarnitines, 21 amino acids, 21 biogenic amines, 76 phosphatidylcholines (PC), 14 lysophosphatidylcholines (lysoPC), 15 sphingomyelins (SM), and 1 sugar) were determined, as described elsewhere^40^ with additional details provided in the AbsoluteIDQ p180 user’s manual (UM_p180_Sciex_13) and the Urine Extension (Biocrates-SOP-p180-Urine) supplement. Quality assurance (QA) and quality control (QC) measures were also implemented as described by Long et al.^40^ We chose to exclude metabolites from analyses with inter-assay coefficients of variation >30%, calculated among pooled QC samples, whose QC status was blinded by lab staff.

The Biocrates Ratio Explorer software calculated 44 biologically relevant metabolite summations and ratios that represent metabolic indicators. Metabolic indicators with sufficient valid data were included in the analysis. To account for urine dilution, metabolite measurements were adjusted to have a constant creatinine concentration (i.e., 1 mmol creatinine). A complete list of the quantified metabolites and metabolic indicator summations and ratios can be found in the Supporting Information (Table S2).

Out of 188 metabolites and 44 metabolic indicators, 10 metabolites and 12 metabolic indicators (that include at least one of the 10 metabolites in their numerator or denominator) were excluded at all time points as they had coefficients of variation >30%. Among those measured with sufficient reliability in QC samples, metabolites and metabolic indicators that were detected in ≥80% of samples (*N*_*T*_1__ = 56, *N*_*T*_2__ = 57, *N*_*T*_3__ = 57) were modeled as continuous variables, metabolites and metabolic indicators detected in between 10 and 79.9% of samples (*N*_*T*_1__ = 94, *N*_*T*_2__ = 91, *N*_*T*_3__ = 95) were modeled as binary variables (detected/not detected), and metabolites and metabolic indicators detected in <10% of samples (*N*_*T*_1__ = 59, *N*_*T*_2__ = 61, *N*_*T*_3__ = 57) were excluded. For endogenous metabolites modeled as continuous variables, values under the limit of detection (LOD) were replaced with the LOD divided by the square root of 2, as detection was high (>80%).^39^ Creatinine was also measured as one of the 188 metabolites but not included in any analysis, as it was used to adjust for urine dilution.

### Covariates

Covariates considered for adjusted models were insurance type (public/private), prepregnancy body mass index (pBMI), maternal age, parity, race/ethnicity, maternal diet quality through the Healthy Eating Index (HEI), tobacco use, and consumption of fruits/vegetables. Race, ethnicity, and tobacco consumption were reported by participants on the questionnaires at enrollment and in follow-up during pregnancy. Race and ethnicity were combined into five categories (i.e., non-Hispanic White, non-Hispanic Black, non-Hispanic Asian, Hispanic, and non-Hispanic other/multiracial). Maternal age, parity, insurance, and weight and height (which were used to calculate pBMI) were obtained through electronic health records at the baseline. The Dietary History Questionnaire II (DHQ-II), a past-year food frequency questionnaire (FFQ), was completed by a subset of participants in our sample (*n* = 594, mean gestational age at questionnaire = 26.86 weeks).^41^

### Statistical Analysis

OP pesticide and metabolomic data were log-transformed to reduce the effect of outliers and to address non-normality of residuals and heteroskedasticity in regression diagnostics.^42^ Metabolomics data was also Pareto scaled due to the high variability in the dynamic ranges of measured metabolites.^43^

Distributions of exposures over the three time points were visualized using box plots (Figure 1) and histograms. To assess the variability of measurements over the three time points, intraclass correlation coefficients (ICCs) were calculated based on a one-way model with a random effect for study participant. Due to interest in time point-specific associations, and since exposures and outcomes were measured in the same urine sample, we estimated associations within each time point (e.g., *T*_1_ OPs and *T*_1_ targeted metabolome, *T*_2_ OPs and *T*_2_ targeted metabolome, etc.) and then assessed robust versus transient associations over the three time points.

**Figure 1:**
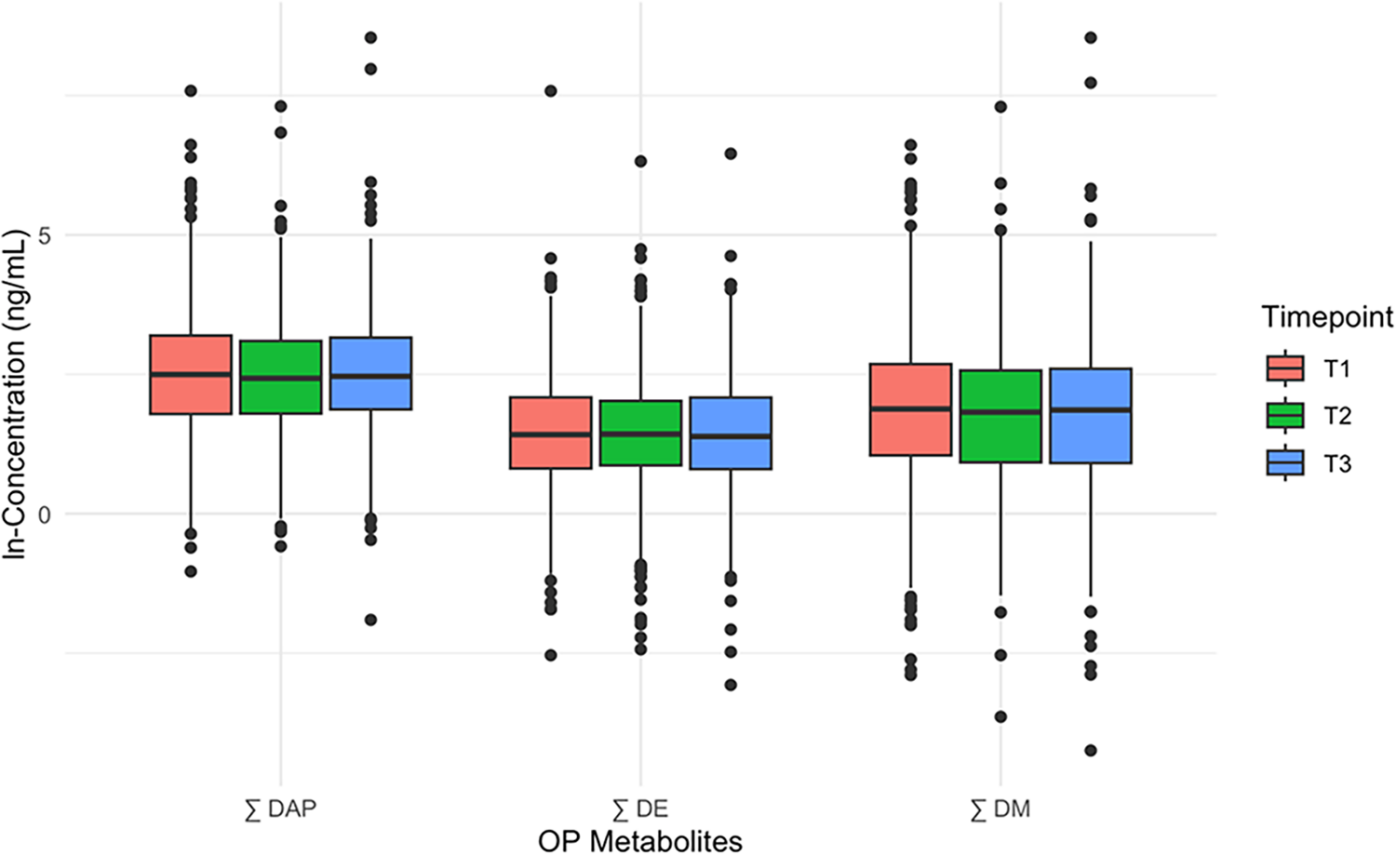
Box plots of the natural log-transformed concentrations of sums of OP pesticide metabolites over time points *T*_1_, *T*_2_, and *T*_3_.

Cross-sectional associations between individual DAPs (DEP, DMP, DETP, DMTP, DMDTP), sums (∑DE, ∑DM, ∑DAP), and individual metabolites and metabolic indicators were estimated using linear regression models for continuous variables, and logistic regression for binary variables, adjusted for potential confounding variables. Additional analyses were conducted modeling exposures as binary variables based on detection frequencies. For exposures with detection frequencies 8–95% (DEDTP, DETP, DMP, DMDTP), exposures were analyzed as “detected” vs “not detected”. For exposures with a detection frequency >95% (DEP, DMTP), exposures were analyzed as “above median” vs “below median”. Log 2 fold changes of endogenous metabolites were calculated between exposure groups, and t test was used to assess statistical significance.

Covariates were selected based on prior literature review, statistical relationships between covariates and exposures, and the construction of a Directed Acyclic Graph (DAG) (Figure S1). The correlation structure of covariates was examined to avoid issues of multicollinearity in adjusted models and to select appropriate proxies among available variables to represent socioeconomic status. The adjusted models included insurance type, maternal age, pBMI, and parity. Race/ethnicity was excluded from the main analysis covariate-adjusted models, as it caused convergence issues due to complete separation and correlation with insurance type, and it was not meaningfully associated with exposure or outcome. However, we conducted a sensitivity analysis including ethnicity (Hispanic/non-Hispanic) in the adjusted models to account for a component of this sociodemographic variable. Additional sensitivity analyses included adjustment for maternal diet quality and the consumption of fruits. While we explored the inclusion of tobacco consumption during pregnancy as a covariate, our study sample did not include enough participants who smoked during or before pregnancy, 1 and 4.4% respectively, to model this relationship. Benjamini-Hochberg false discovery rate (FDR) correction (5%) was applied to account for multiple hypothesis testing among each time point and type of model (continuous or binary), as is the common approach in omics analyses.^44–46^ Single imputation was applied for missing covariate data, as no covariate was missing >1% of observations.

## Results

### Study Population Characteristics

Demographic and health characteristics for the study population, by low vs high total average exposure (where “low” exposure refers to below the median concentration of the average of ∑DAP across available time points, and “high” refers to above the median), are shown in Table 1. The mean gestational age at urine collection was 10.78 weeks (standard deviation (SD) = 3.10 weeks), 20.89 weeks (SD = 1.84 weeks), and 29.48 weeks (SD = 3.54 weeks) for *T*_1_, *T*_2_, and *T*_3_, respectively.

**Table 1:** Demographic Characteristics of the Study Sample by Exposure Level^a^,^b^

	high exposure >13.78 ng/mL (*n* = 445)	low exposure <13.78 ng/mL (*n* = 445)
age mean (SD)	32.0 (5.4)	31.2 (5.8)
pBMI mean (SD)	25.5 (5.5)	27.0 (5.8)
race/ethnicity		
Hispanic	195 (43.9%)	270 (60.9%)
non-Hispanic White	170 (38.3%)	106 (23.9%)
non-Hispanic Black	18 (4.1%)	33 (7.4%)
Asian	42 (9.5%)	28 (6.3%)
other/multiple	19 (4.3%)	26 (1.4%)
parity		
nulliparous	230 (51.8%)	205 (46.3%)
parous	214 (48.2%)	238 (53.7%)
insurance type		
public	227 (51.0%)	264 (59.3%)
private	218 (49.0%)	181 (40.7%)
gestational diabetes		
no	387 (87.0%)	378 (84.9%)
yes	58 (13.0%)	67 (15.1%)
education status		
high school or less	144 (33.7%)	171 (39.3%)
some college	62 (14.5%)	79 (18.1%)
bachelor’s degree	102 (23.9%)	98 (22.5%)
postgraduate degree	119 (27.9%)	87 (20.0%)

a Missing; pBMI = 2, Race/ethnicity = 3, Parity = 3, Education = 28.

b “Low” exposure refers to below the median concentration of the average of ∑DAP across available time points, and “high” refers to above the median.

### Exposure Prevalence

All DAPs included in the analysis were detected in >80% of samples. The mean concentrations of ∑DAP were 29.5 ng/mL (SD = 96.6 ng/mL), 23.6 ng/mL (SD = 74.7 ng/mL), and 33.9 (238.7 ng/mL) for *T*_1_, *T*_2_, and T_3_, respectively. The mean and standard deviations of concentrations of the other exposure variables are shown in Table 2, and the distributions of the ∑DAP, ∑DE, and ∑DM are displayed in Figure 1. The ICC for the log-transformed ∑DAP across the time points was 0.23, indicating concentrations were unstable over time within individuals.

**Table 2:** Mean (SD) Concentrations (ng/mL) of Urinary OP Metabolites by Timepoint^a^

	∑DAP	∑DE	∑DM	DMP	DEP	DMTP	DETP	DMDTP
*T*_1_	29.51 (96.56)	10.39 (79.77)	19.12 (53.93)	5.91 (12.55)	8.57 (79.46)	9.03 (30.82)	1.49 (4.53)	4.18 (25.70)
*T*_2_	23.58 (74.70)	7.88 (24.39)	15.69 (64.73)	5.22 (11.70)	6.06 (22.40)	8.14 (51.63)	1.44 (3.73)	2.34 (9.72)
*T*_3_	33.88 (238.73)	8.13 (27.23)	25.75 (226.88)	6.10 (26.57)	6.07 (15.98)	9.08 (66.14)	1.73 (11.62)	10.57 (204.43)

a ∑DAP = mass sum of all dialkyl phosphate metabolites; ∑DEP = mass sum of all diethyl phosphates; ∑DMP = mass sum of all dimethyl phosphates; DMP = dimethyl phosphate; DEP = diethyl phosphate; DMTP = dimethyl thiophosphate; DETP = diethyl thiophosphate; DMDTP dimethyl dithiophosphate.

### Metabolite Associations with OP Pesticide Exposure

#### Overall Associations

152 metabolites and metabolic indicators were detected at a frequency sufficient in at least one time point to be included in any model. Among these metabolites, there were 38 acylcarnitines, 14 amino acids, 17 biogenic amines, 10 lysophosphatidylcholines, 52 phosphatidylcholines, 14 sphingomyelins, 1 monosaccharide, and 7 summations or ratios that represent metabolic indicators. A total of 81/152 metabolites and metabolic indicators were statistically significantly associated with at least one exposure during one time point in the raw models after FDR correction, while in the covariate-adjusted models, this number was reduced to 59/152.

There was consistency for which metabolites were significant hits across exposures, particularly among adjusted models. Most metabolites that were associated with at least one of eight exposures were significantly associated with more than one exposure (70 and 78%, respectively, for raw and adjusted models). Most (75%, 60/81 for raw and 44/59 for adjusted) were also significantly associated with at least one of the mass sums (∑DAP, ∑DE, or ∑DM), suggesting these sums captured relevant variation in exposure levels. Figures 2, 3, and 4 highlight results from covariate-adjusted models for ∑DAP, ∑DE, and ∑DM exposures. Tables with outputs for all models can be found in the Supporting Information (Tables S3 and S4).

**Figure 2:**
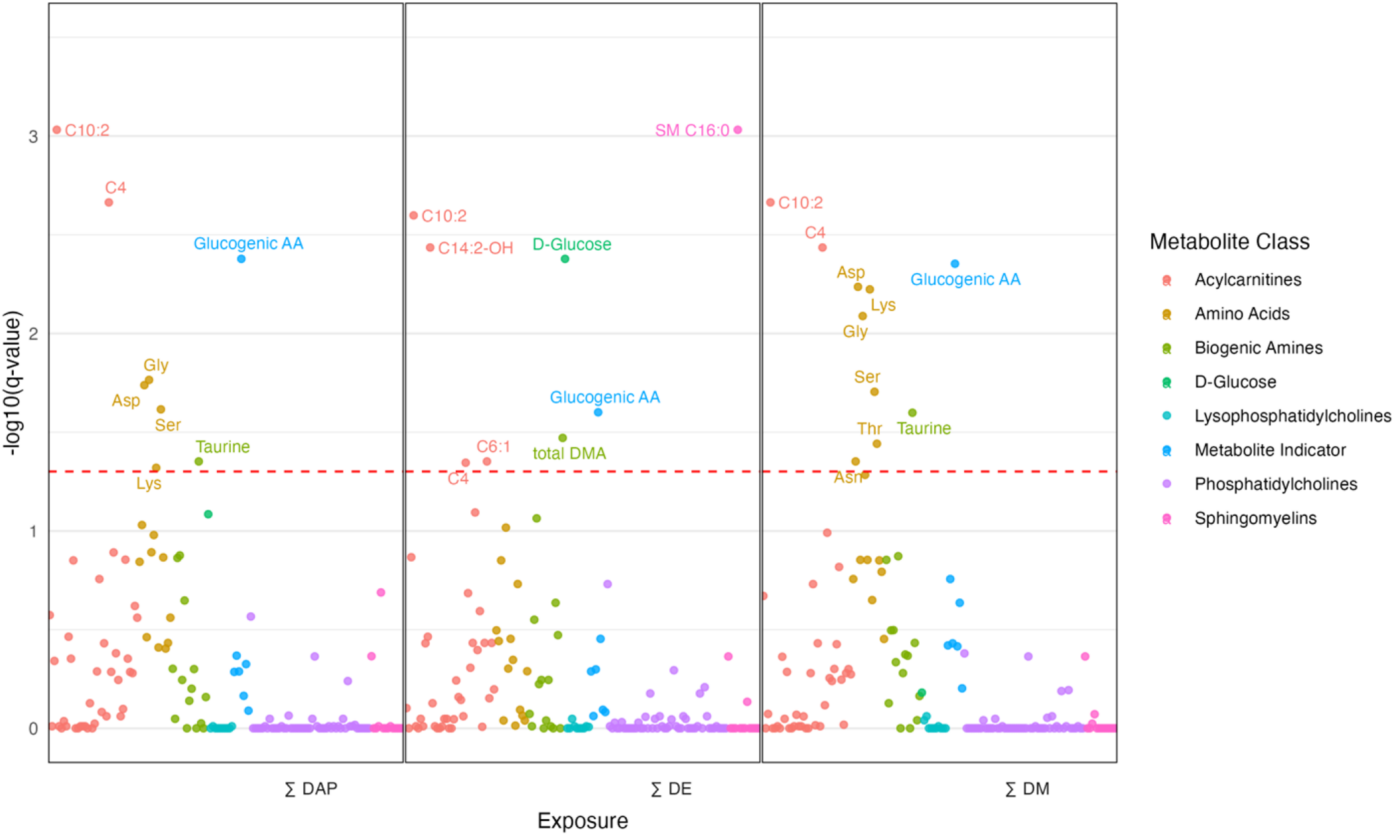
Manhattan plot of early pregnancy (*T*_1_) associations between sums of OP pesticide metabolites and endogenous metabolites adjusted for covariates (pBMI, insurance type, age, parity). Significant metabolites are labeled. The red dotted line represents the FDR-adjusted *p*-value = 0.05.

**Figure 3:**
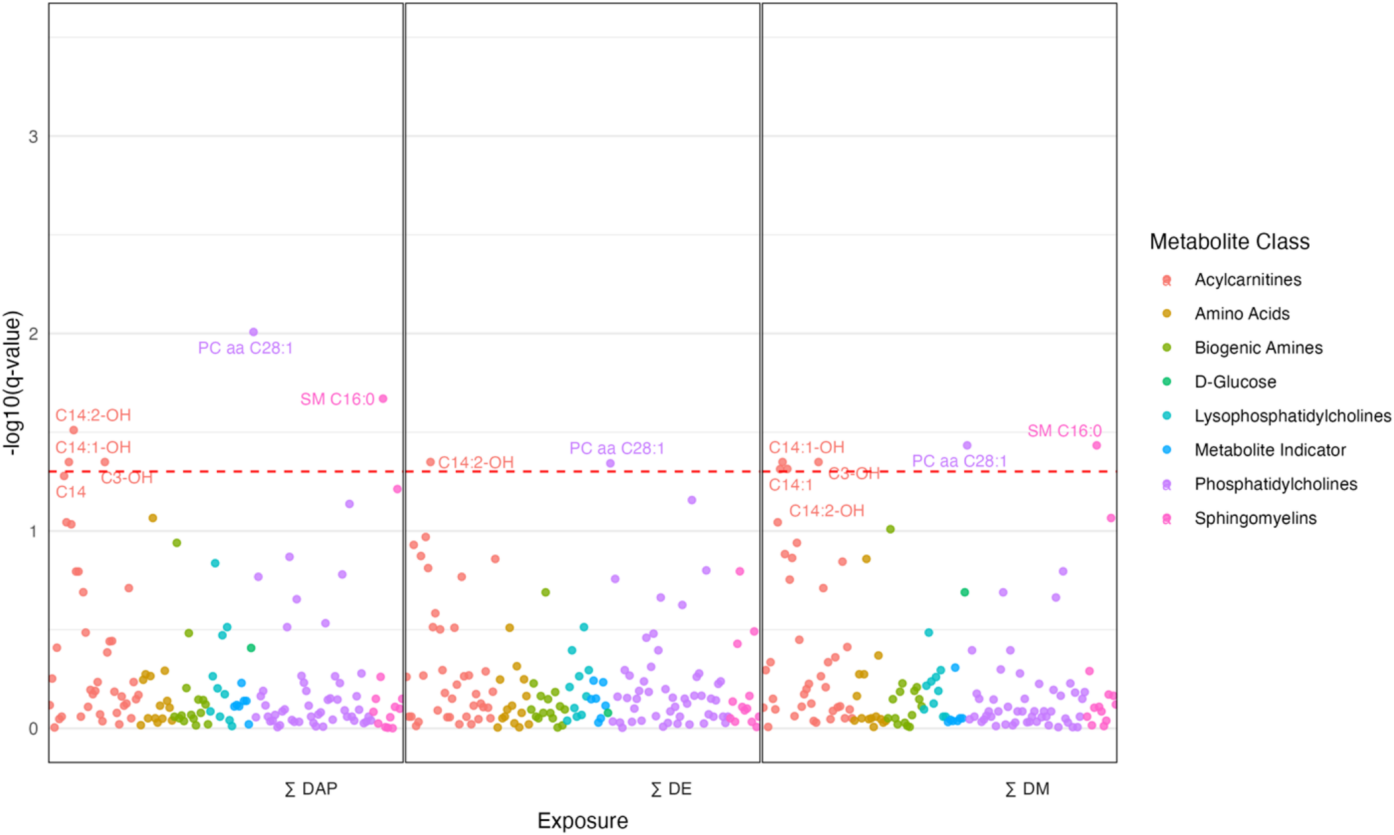
Manhattan plot of midpregnancy (*T*_2_) associations between sums of OP pesticide metabolites and endogenous metabolites adjusted for covariates (pBMI, insurance type, age, parity). Significant metabolites are labeled. The red dotted line represents the FDR-adjusted *p*-value = 0.05.

**Figure 4:**
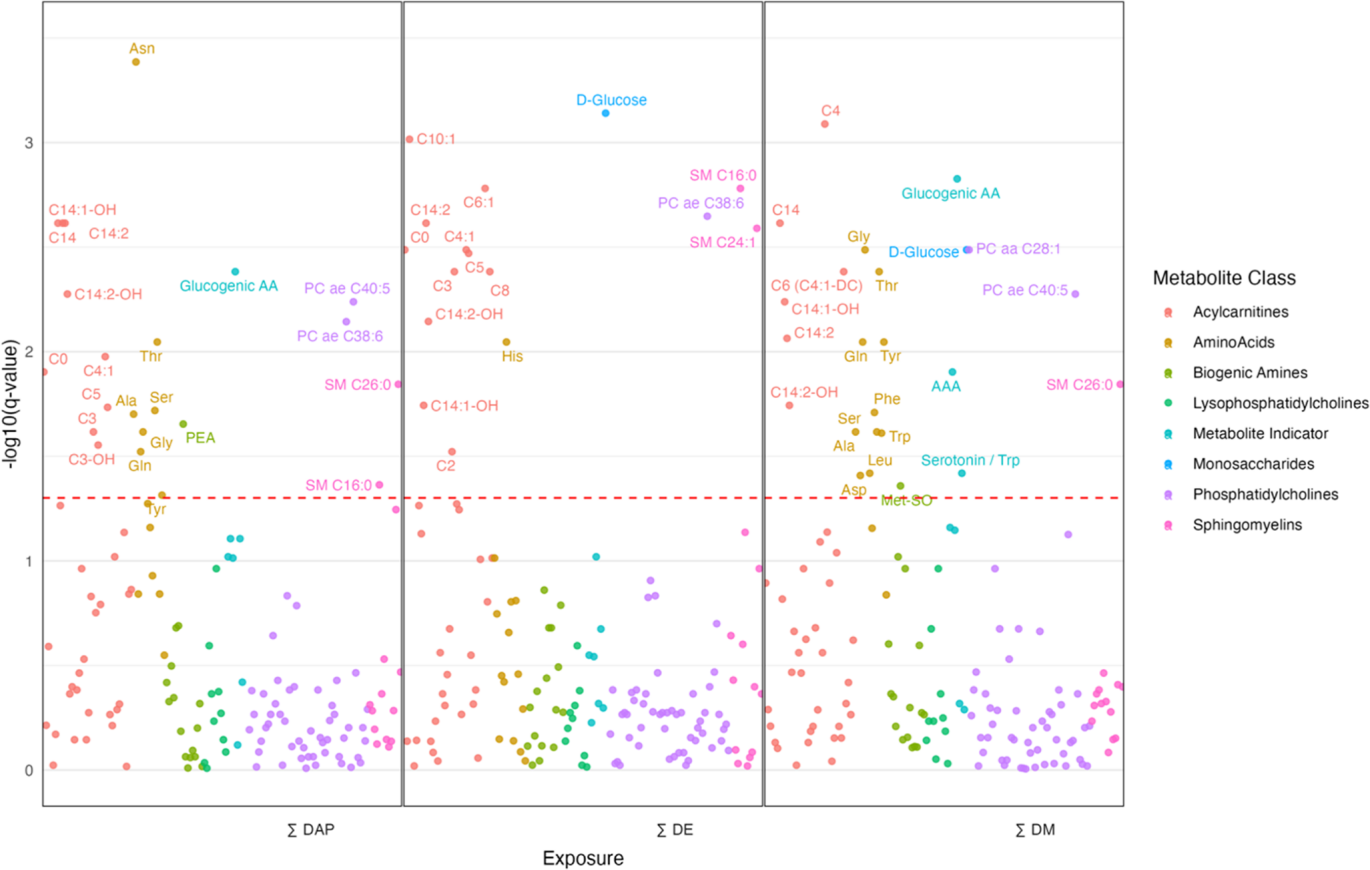
Manhattan plot of late pregnancy (*T*_3_) associations between sums of OP pesticide metabolites and endogenous metabolites adjusted for covariates (pBMI, insurance type, age, and parity). Significant metabolites are labeled. The red dotted line represents the FDR-adjusted *p*-value = 0.05.

Among covariate-adjusted models, 44/152 distinct metabolites and metabolic indicators were significantly associated with ∑DAP, ∑DE, and ∑DM exposures. The breakdown of metabolites by class that were measured, analyzed, and significantly associated with exposure in robust models and covariate-adjusted models using summed quantities as exposure in any time point are shown in Table 3. There was modest consistency in what metabolites were significant over each time point. For robust models, 28/44 were significant in one time point only, 14/44 were significant in two time points, and 2/44 were significant in all three. This demonstrates that time point-specific associations are present.

**Table 3:** Breakdown of Endogenous Metabolites by Class That Were Measured, Analyzed, and Significantly Associated with Exposure in Robust Models in Any Timepoint

metabolite class	# measured^a^	# modeled^b^	# significant^c^	% significant^d^
acylcarnitines	40	38	17	45
amino acids	21	14	13	93
biogenic amines	21	16	4	25
phosphatidylcholines	76	52	3	6
lysophosphatidylcholines	14	10	0	0
sphingmyelins	15	14	3	21
sugars	1	1	1	100
metabolic indicators (sums/ratios)	44	7	3	43
total	232	152	44	29

a Measured = endogenous metabolites covered in the Biocrates p180 targeted metabolomics assay.

b Modeled = endogenous metabolites with sufficient data available to be included in binary or continuous models.

c Significant = endogenous metabolites significantly associated with at least 1 of: ∑DAP, ∑DE, or ∑DM in covariate-adjusted models.

d % Significant = percentage of metabolites that were included in binary or continuous model that were significantly associated with at least 1 of: ∑DAP, ∑DE, or ∑DM in covariate-adjusted models.

### *T*_1_, Early Pregnancy, <18 Weeks

Among the 150 metabolites and indicators analyzed in *T*_1_, 56 were treated as continuous variables and 94 were treated as binary. In unadjusted analyses, 22 metabolites were significantly associated with at least one exposure (∑DAP, ∑DE, or ∑DM), which reduced to 15 after covariate adjustment (Figure 2). Most (8/15) were linked to two or more exposures. Acylcarnitines C10:2 and C4, and the glucogenic amino acids, were associated with all three exposures. Effect estimates for significant endogenous metabolites were small in magnitude ranging from −0.09–0.16 and mostly positive, with a 1% exposure increase corresponding to a 0.6–0.15% rise in metabolite levels, except for dimethylamine (DMA), which showed a −0.1% decrease. Other significant metabolites included serine, lysine, asparagine, glycine, threonine, biogenic amines C14:2-OH and taurine, sphingomyelin SM C16:0, and hexose. No phosphatidylcholines or lysophosphatidylcholines were significantly associated with OP pesticide exposure in *T*_1_ samples.

### *T*_2_, Midpregnancy, 18–25 Weeks

In the *T*_2_ analysis, 148 metabolites and indicators were included, with 57 analyzed as continuous variables and 91 analyzed as binary. Unadjusted analyses identified 28 metabolites significantly associated with at least one exposure (∑DAP, ∑DE, or ∑DM), which decreased to 7 after covariate adjustment (Figure 3). These included acylcarnitines (C14, C14:1, C14:1–OH, C14:2-OH, C3–OH), sphingomyelin (SM C16:0), and phosphatidylcholine (PC aa C28:1). Acylcarnitines were analyzed as binary variables, while sphingomyelin and phosphatidylcholine were continuous. C14 and C14:1 were linked to one exposure (∑DAP, ∑DE, or ∑DM), C14:1–OH, C3–OH, and SM C16:0 to two, and C14:2-OH and PC aa C28:1 to all three.

In binary models, effect estimates were all below one (0.70–0.81), indicating a 19–30% decrease in detection odds per unit increase in log-transformed exposure. Continuous models showed positive associations (0.08 to 0.14), with a 1% exposure increase corresponding to an 0.8 to 0.14% rise in standardized metabolite levels (Figure 3).

### *T*_3_, Late Pregnancy, 18–25 Weeks

In the *T*_3_ analysis, 152 metabolites and indicators were included, with 57 analyzed as continuous variables and 95 as binary. Unadjusted models identified 45 metabolites significantly associated with at least one exposure (∑DAP, ∑DE, or ∑DM), which decreased to 40 after the covariate adjustment (Figure 4). These included 16 acylcarnitines, 12 amino acids, 2 biogenic amines, 3 phosphatidylcholines, 3 sphingomyelins, 3 metabolic indicators, and 1 sugar. Of the 40 significant metabolites, 14 were linked to one exposure (∑DAP, ∑DE, or ∑DM), 17 to two, and 9 to all three. In binary models, most effect estimates were below one (0.71–0.8), indicating a 20–29% decrease in detection odds per unit increase in log-transformed exposure. The exception was PC ae 40:5, which showed a 26 and 32% decrease for ∑DAP and ∑DM, respectively. In continuous models, effect estimates ranged from 0.06–0.20, reflecting a 0.06–0.20% increase in standardized metabolite levels per 1% exposure increase, except for the serotonin/tryptophan ratio, which decreased by 0.05%.

### Sensitivity Analyses

Sensitivity analyses were conducted to compare with the *T*_3_ main analyses, where the most significant associations were observed. Full model results are in the Supporting Information (Tables S5–S7). Including ethnicity in the models had no impact on findings—the same 40 metabolites remained significant, with effect estimates highly correlated (*r* = 0.998). When accounting for diet, results were moderately robust. Including diet fruit density reduced significant metabolites from 40 to 20, and adding the HEI score further reduced this to 17. This reduction, however, likely reflects decreased power due to a smaller sample size (768 in the main analysis vs 594 with DHQ data). This is supported by consistent effect estimates despite increased *p*-values, no new significant metabolites, and the same metabolites losing significance in both diet models. Effect estimates remained highly correlated with the main analysis (*r* = 0.91), and no association changed direction in any model. Similar results were also observed when exposures were analyzed as binary variables. Exposure was statistically significantly related to a higher number of altered endogenous metabolites compared to the main analysis, particularly phosphatidylcholines and sphingomyelins, perhaps due to the inclusion of DEDTP, and modeling of all endogenous metabolites as continuous variables. However, the overall trends remain the same, with more metabolites being elevated versus decreased with respect to exposure (Figure 5). All t-test results from this analysis can be found in the Supporting Information (Table S8).

**Figure 5:**
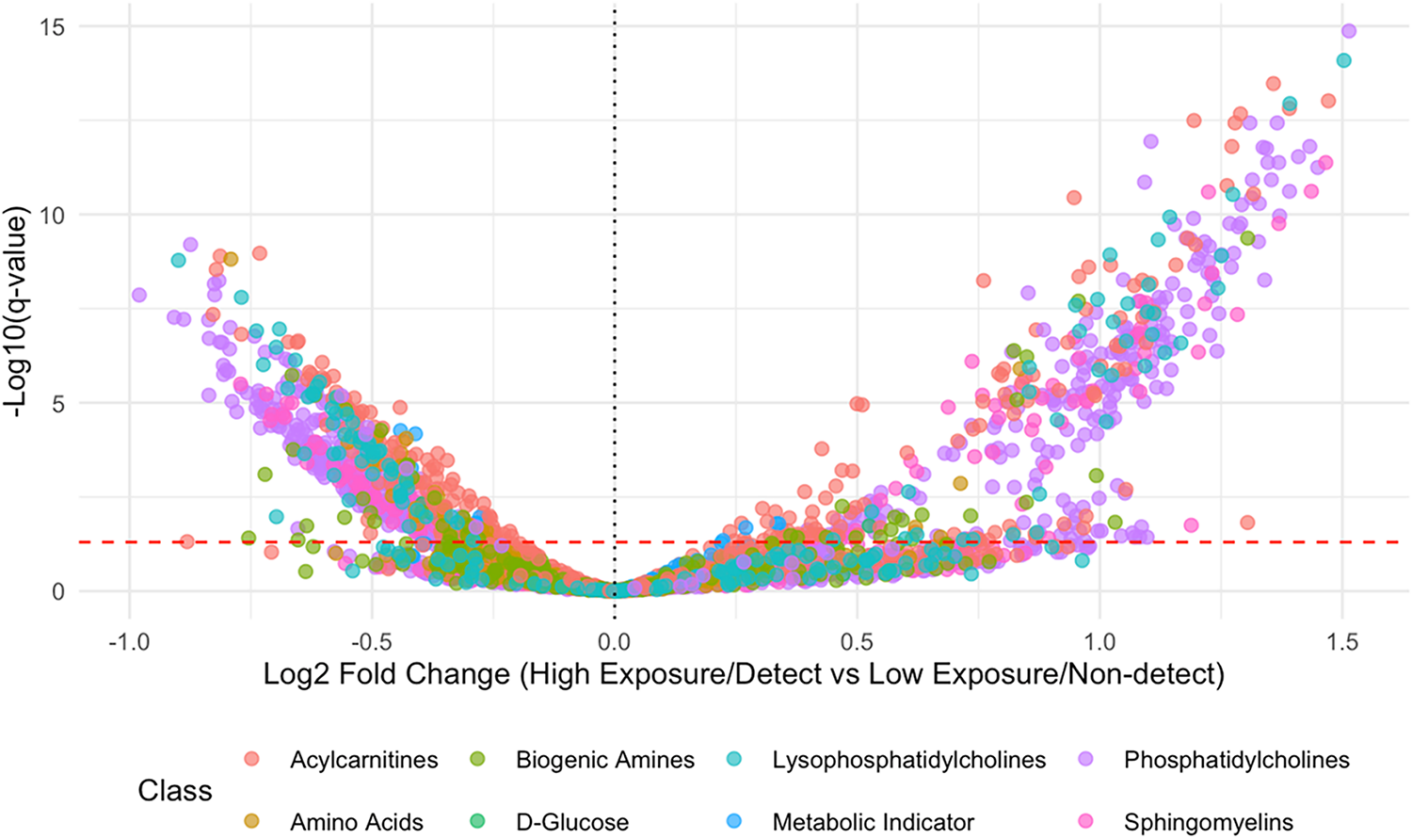
Volcano plot of differences in endogenous metabolites between exposure groups. Due to detection frequencies, DEDTP, DETP, DMP, and DMDTP were analyzed as “detected” vs “not detected” and DEP and DMTP were analyzed as “high exposure” vs “low exposure”, with the threshold for “high exposure” being above the median exposure value.

## Discussion

### Summary of Key Findings

This study aimed to investigate the associations between prenatal organophosphate (OP) pesticide exposure and maternal metabolic profiles during pregnancy in the NYU CHES cohort. We identified significant associations between OP exposure and targeted endogenous metabolites across several classes, including acylcarnitines, phosphatidylcholines, sphingomyelins, amino acids, hexoses, and biogenic amines. In early pregnancy (<18 weeks), increased urinary biomarkers of OP exposure were significantly associated with increased concentrations of acylcarnitines (C10:2, C4, C14:3–OH, and C6:1), amino acids (aspartic acid, asparagine, glycine, histidine, serine, threonine, and overall glucogenic amino acids), sphingomyelin (SM C16:0), and d-Glucose. Additionally, a significant decrease in dimethylamine (DMA) concentrations was observed. During midpregnancy (18–25 weeks), increased OP pesticide exposure was associated with increased concentrations of phosphatidylcholine (PC aa C28:1) and sphingomyelin (SM C16:0), along with decreased odds of detecting acylcarnitines (C14, C14:1, C14:1-OH, and C14:2-OH). In late pregnancy (>25 weeks), a broader spectrum of metabolite alterations was observed. Higher exposure was linked to increased concentrations (C0, C10:1, C10:2, C2, C3, C4, C4:1, C5, C6, C6:1, and C8) or lower odds of detection (C14, C14:1–OH, C14:2, C3–OH) of multiple acylcarnitines. Amino acid concentrations were also elevated, including alanine, aspartic acid, asparagine, glutamine, glycine, histidine, leucine, phenylalanine, serine, threonine, tryptophan, tyrosine, and glucogenic amino acids overall. Increased concentrations of biogenic amine methionine-sulfoxide (Met-SO) and decreased odds of detection of biogenic amine PEA were also noted. Additionally, corresponding to increased exposure, there were increased concentrations of sphingomyelins (SM C16:0, SM C24:1, and SM C26:0) and phosphatidylcholines (PC ae C38:6, PC ae C28:1) while the odds of detecting phosphatidylcholine (PC ae C40:5) were increased. Further, increased concentration of the d-Glucose was observed, along with a decreased ratio of serotonin to tryptophan. No associations were documented between exposure and lysophosphatidylcholines at any time point.

These findings suggest that organophosphate pesticide exposure during pregnancy may have trimester-specific metabolic effects, with the most extensive changes occurring in late pregnancy. The fewest associations between exposure and metabolites occurred during midpregnancy, although this could be somewhat due to the smaller sample size in this time point. To the best of our knowledge, this is the first study investigating the associations between OP exposure measured as urine DAP metabolites and targeted urine metabolomics during pregnancy.

### Pathways of Toxicity

#### Oxidative Stress and Mitochondrial Dysfunction

Two related suggested mechanisms of OP pesticide toxicity with significant evidence are oxidative stress^5,8,25,26,47–50^ and mitochondrial dysfunction.^7,24,51^ Both are thoroughly related to lipid metabolism and redox balance and can affect or be indicated by various metabolic pathways.

Modest evidence of oxidative stress related to OP exposure was found in this study. In *T*_3_, observed concentrations of Met-SO, a potential indicator of oxidative stress,^52^ was increased among participants with increased exposure to OP pesticides. However, there was no significant association with oxidation biomarker α-aminoadipic acid (α-AAA).^53,54^ Indirect measures that could loosely suggest but are not specific to oxidative stress, such as altered amino acid and lipid metabolism, were also observed.

There was stronger evidence to support mitochondrial dysfunction as a valid mechanism in this study. Elevated concentrations of short and medium-chain acylcarnitines in *T*_1_ and *T*_3_ were consistently observed in relation to increased DAP concentration across exposures and in sensitivity analyses. Longer chain acylcarnitines were depressed in *T*_2_ and *T*_3_. Additionally, the metabolic indicator representing the measure of overall β-oxidation activity, (C2 + C3)/C0,^55,56^ was consistently inversely associated with increased exposure, for all exposures and time points. When the FDR adjustment was applied to *p*-values, this association was significant only for DMDTP at *T*_3_, though other exposures remained close to the significance threshold (Tables S3 and S4), providing support for the hypothesized mechanism.

Acylcarnitines play a crucial role in β oxidation of fatty acids, and altered urine levels indicate mitochondrial dysfunction and are often even used as a screening and diagnostic tool to identify disorders of fatty acid oxidation.^57,58^ While other studies in humans have not specifically studied acylcarnitine profiles in relation to DAP metabolite concentrations in pregnancy, several studies in humans, animals, and in vitro, provide substantial evidence that exposure to organophosphate pesticides and other environmental pollutants can trigger mitochondrial dysfunction,^59–62^ is consistently related to altered fatty acid oxidation,^63,64,65^ and can lead to an accumulation of and thus increased excretion of acylcarnitines.^66,67^ Some manifestations of mitochondrial dysfunctions can also lead to decreased acylcarnitines, particularly of long chains, which was observed in our study.^57,58,68^

An untargeted metabolomics study looking at metabolomic differences related to OP exposure in adults in the Central California Valley, found similar results—exposure was consistently related to acylcarnitines (e.g., C8, C12) and pathways involved in the carnitine shuttle and β oxidation.^64^ While they report increased exposure was significantly associated with decreased concentrations of medium-chain acylcarnitines, this could represent differences in the biological matrix that was analyzed (serum vs urine).^66^ Similarly, an untargeted metabolomics analysis of serum of pregnant farm workers exposed to chlorpyrifos found many significant associations with metabolites involved in fatty acid oxidation, including the acyl-CoA precursors to acylcarnitines.^63^ Disrupted acylcarnitine profiles have also been associated with phenotypes and diseases potentially associated with OP exposure, such as ASD.^69^ Serine, an amino acid, was observed in association with OP exposure in *T*_1_ and *T*_3_, which has significant implications on mitochondrial function.^70^

#### Inflammation/Immune System Activation

Another key proposed mechanism of OP pesticide toxicity is immune system activation and inflammation.^14,47^ In our study, this pathway was indirectly probed via analyzing concentrations of molecules involved in lipid metabolism: sphingomyelins and phosphatidylcholines. These metabolites play important roles in membrane structure and function, cell signaling, and even placental vasculature during pregnancy^71–73^ and disruptions to their homeostasis have been associated with inflammation.^74,75^ Disrupted profiles of sphingolipids have been significantly associated with exposure to organophosphate flame retardants, a class of compounds structurally related to OP pesticides.^76^ Both sphingolipids and phosphatidylcholines have been associated with metabolic pregnancy complications including gestational diabetes and preeclampsia^71,77,78^ and can indicate maternal immune activation/inflammation.^79,80^ Maternal immune activation is also shown to disrupt normal metabolic processes and alter lipid, amino acid, and nucleotide metabolism. Additionally, in this study, the metabolic indicator of the rate of degradation of tryptophan to serotonin (Serotonin/Trp) was significantly decreased in relation to increased OP exposure in *T*_3_ (Figure 4). This could be caused by diversion of tryptophan to the kynurenine pathway, which happens under conditions of stress and inflammation.^81,82^ Thus, observations of these altered processes in our study generally provide further, albeit indirect evidence for this pathway.^83^

#### Amino Acid Metabolism and Related Pathways

Of the metabolites included in the analysis, amino acids have been studied the most with respect to OP pesticide exposure. Amino acids are fundamental building blocks of proteins and play crucial roles in various metabolic pathways, Clinically, disruptions in amino acid metabolism are linked to metabolic disorders, neurological diseases, and hepatic abnormalities, etc.^84^ While not exhaustive, among the most consistently reported associations between OP exposure and amino acids include alanine, tryptophan, glutamine, glutamine glycine, cysteine, phenylalanine, and threonine; all of which were significant in our study in *T*_1_, *T*_3_, or both, with the exception of cysteine and glutamate.^61–65,85–89^ Additionally, increased exposure was associated with increased concentrations of the metabolic indicator of glycolytic to gluconeogenesis activity (glucogenic AA’s) in *T*_1_ and *T*_3_. Elevated gluconeogenesis can be caused by oxidative stress and mitochondrial dysfunction, and increasing evidence suggests this may be an important step on the path to insulin resistance in diabetes mellitus.^90–92^

Increased tryptophan and glutamine, as well as a decreased serotonin to tryptophan ratio, a metabolic indicator of the rate of tryptophan degradation, were significantly associated with increased DAP levels in our cohort in late pregnancy. These metabolites are critical precursors of neurotransmitters and important for the serotonin-tryptophan, tryptophan-kynurenine, and glutamate-GABA pathways, respectively, all significant proposed mechanisms of OP pesticide neurotoxicity.^8,11,14,47,84,93^

#### Strengths and Limitations–Future Directions

Our study benefits from a large sample of nonoccupationally exposed pregnant participants with measures of exposure and targeted metabolites throughout pregnancy, providing us the opportunity to detect time point-specific effects during sensitive periods at exposure levels relevant to the general population. Additionally, we were able to adjust for multiple confounders, and findings were robust, counteracting typical trends in a pregnant healthy population and suggesting metabolite differences beyond what is expected throughout pregnancy.^94^ Unlike many studies, we have rich diet history questionnaire data to draw from to consider diet quality and fruit density in diet as covariates, which are important determinants of both pesticide exposure and the metabolism.^32^

Limitations include relying on single-spot urine samples to assess OP exposure, which may not fully capture the long-term exposure variability. Additionally, we do not have information on specific OP compounds, and findings may have limited generalizability to other populations. These analyses are also cross-sectional, so it is difficult to disentangle cause and effect, and reverse causation cannot be ruled out. DHQ data is subject to inaccuracy and lack of specificity in timing as it reflects total past-year diet habits, instead of a more recent food diary, which would be more desirable. Additionally, we do not have access to specific gravity data and thus relied on creatinine to account for urine dilution. This adjustment—especially when applied during pregnancy when physiological changes in renal function are occurring—is not favorable and contributes to intraindividual variability and systematic variation.^95^ Future studies should include adjustment for urine specific gravity, investigate the specific OP compounds contributing to the observed metabolic changes, and aim to further understand the functional significance and health implications of metabolic alterations. It would also be valuable to replicate these findings in other populations and biosamples (e.g., serum) and to examine the potential for interventions to mitigate the effects of OP exposure.

Our study showed significant, time point-specific alterations of urinary metabolites during pregnancy associated with increased concentrations of DAP metabolites. We focus on presenting the most robust findings of metabolites associated with sums of DAPs (∑DAP, ∑DE, and ∑DM) after adjustment for multiple potential confounding variables and FDR. Significant relationships between OP exposure and metabolites involved in amino acid and lipid metabolism were consistently observed. Particularly, acylcarnitine profiles were altered, suggesting mitochondrial dysfunction as an important mechanism of OP induced toxicity

## Supplementary Material





## Notes

Demographic characteristics of the entire NYU CHES cohort vs the study sample; list of metabolites measured by Biocrates AbsoluteIDQ p180; metabolic indicators from Biocrates Ratio Explorer software; Directed Acyclic Graph; results for unadjusted analysis; results for covariate-adjusted analysis; results for sensitivity analyses, including HEI total score as covariate; results for sensitivity analyses, including fruit density total score as covariate; results for sensitivity analyses, including ethnicity as covariate (Table S1 and Figure S1); Biocrates manual (PDF)
Metabolites; metabolic indicators; results from covariate-adjusted analyses; results from covariate-adjusted analyses; results from sensitivity analysis, including diet fruit density as a covariate, Timepoint = *T*_3_; results from sensitivity analysis, including ethnicity as a covariate, Timepoint = *T*_3_ (XLSX)
