# Supplementary material for: Prenatal Organophosphate Pesticide Exposure and Targeted Maternal Pregnancy Metabolomic Profiles in the NYU CHES Cohort
Source: Environ Sci Technol. 2025 Oct 10;59(41):21848–59. doi: 10.1021/acs.est.5c05412 (PMC12550812; doi:10.1021/acs.est.5c05412)
Supplement: Supplementary file 1 [file es5c05412_si_001.pdf]

Prenatal organophosphate pesticide exposure and targeted maternal pregnancy metabolomic profiles in the NYU CHES cohort

Authors
Haleigh Cavalier[1, 2*], Akhgar Ghassabian[1,2], Sara E. Long[2], Yelena Afanasyeva[1,2], Susan Sumner[3,4], Susan McRitchie[4], Rachel Coble[4], Yu Chen,[1] Kurunthachalam Kannan[5], Zhongmin Li[5], Mengling Liu[1], Leonardo Trasande[1,2,6]

Affiliations
[1]Department of Population Health, NYU Langone Medical Center, New York, NY, 10016, USA
[2]Department of Pediatrics, Division of Environmental Pediatrics, NYU Langone Medical Center, New York, NY,10016, USA
[3]Department of Nutrition, UNC Chapel Hill, Chapel Hill, NC, 27516, USA
[4]UNC Chapel Hill Nutrition Research Institute, Kannapolis, NC, 28081, USA
[5]Wadsworth Center, New York State Department of Health, Albany, NY, 12237, USA
[6]NYU Wagner School of Public Service, New York, NY, 10003 USA

*corresponding author, email: haleigh.cavalier@nyulangone.org

Table S1. Demographic characteristics of entire NYU CHES cohort vs study sample
Table S2. A) Metabolites measured by Biocrates AbsoluteIDQ p180 assay B) Metabolic indicators from Biocrates Ratio Explorer software, “Table S2.xlsx”
Figure S1. Directed Acyclic Graph
Tables S3. Results for unadjusted analysis
Tables S4. Results for covariate-adjusted analysis
Tables S5. Results for sensitivity analyses, including HEI total score as covariate
Table S6. Results for sensitivity analyses, including fruit density total score as covariate
Table S7. Results for sensitivity analyses, including ethnicity as covariate
Table S8. Results for sensitivity analysis, binary exposure variables

	**NYU CHES (N=3549)**	**Study Sample (N=890)**	**Total (N=4439)**
**Hospital Site of Recruitment**			
Bellevue	267 (7.5%)	232 (26.1%)	499 (11.2%)
NYU Brooklyn	1317 (37.1%)	260 (29.2%)	1577 (35.5%)
NYU Manhattan	1933 (54.5%)	398 (44.7%)	2331 (52.5%)
Other	32 (0.9%)	0 (0.0%)	32 (0.7%)
**Pregnancy Outcome**			
Live Birth	3140 (88.5%)	861 (96.7%)	4001 (90.1%)
Stillbirth	18 (0.5%)	2 (0.2%)	20 (0.5%)
Elective Termination	65 (1.8%)	5 (0.6%)	70 (1.6%)
Miscarriage	199 (5.6%)	14 (1.6%)	213 (4.8%)
Pregnant	0 (0.0%)	0 (0.0%)	0 (0.0%)
Unknown	127 (3.6%)	8 (0.9%)	135 (3.0%)
**Race/Ethnicity**			

	NYU CHES (N=3549)	Study Sample (N=890)	Total (N=4439)
Hispanic	1580 (46.3%)	465 (52.4%)	2045 (47.6%)
Non-Hispanic White	1209 (35.4%)	276 (31.1%)	1485 (34.5%)
Non-Hispanic Black	199 (5.8%)	51 (5.7%)	250 (5.8%)
Asian	318 (9.3%)	70 (7.9%)	388 (9.0%)
Other/Multiple	107 (3.1%)	25 (2.8%)	132 (3.1%)
Missing	136	3	139
**BMI**			
Mean (SD)	26.495 (5.871)	26.210 (5.670)	26.436 (5.830)
**Age**			
Mean (SD)	32.267 (5.584)	31.586 (5.625)	32.131 (5.598)
**Employment Status**			
N-Miss	307	14	321
No	1124 (34.7%)	323 (36.9%)	1447 (35.1%)
Yes	2118 (65.3%)	553 (63.1%)	2671 (64.9%)
**Parity**			
Nulliparous	1645 (46.7%)	435 (49.0%)	2080 (47.2%)
Parous	1878 (53.3%)	452 (51.0%)	2330 (52.8%)
Missing	26	3	29
**Insurance Type**			
Public	1617 (46.4%)	491 (55.2%)	2108 (48.2%)
Private	1865 (53.6%)	399 (44.8%)	2264 (51.8%)
Missing	67	0	67
**Tobacco consumption during pregnancy**			
No	2943 (98.7%)	860 (99.0%)	3803 (98.8%)
Yes	39 (1.3%)	9 (1.0%)	48 (1.2%)
Missing	567	21	588
**Alcohol consumption during pregnancy**			
No	2527 (85.2%)	757 (87.2%)	3284 (85.7%)
Yes	439 (14.8%)	111 (12.8%)	550 (14.3%)
Missing	583	22	605
**Marital Status**			
Married/living with a partner	2834 (85.6%)	776 (87.9%)	3610 (86.1%)
Divorced/separated	83 (2.5%)	14 (1.6%)	97 (2.3%)
Single/widowed	393 (11.9%)	93 (10.5%)	486 (11.6%)
Missing	239	7	246
**Household Income**			
Less than $30,000	447 (15.9%)	146 (17.5%)	593 (16.2%)

	NYU CHES (N=3549)	Study Sample (N=890)	Total (N=4439)
$30,000 - $49,999	188 (6.7%)	81 (9.7%)	269 (7.4%)
$50,000 - $99,999	293 (10.4%)	101 (12.1%)	394 (10.8%)
$100,000 or more	1300 (46.1%)	271 (32.5%)	1571 (43.0%)
Don't Know	590 (20.9%)	235 (28.2%)	825 (22.6%)
Missing	731	56	787
**Education Status**			
High school or less	876 (30.1%)	315 (36.5%)	1191 (31.6%)
Some college	416 (14.3%)	141 (16.4%)	557 (14.8%)
Bachelor's degree	695 (23.9%)	200 (23.2%)	895 (23.7%)
Post-graduate degree	924 (31.7%)	206 (23.9%)	1130 (29.9%)
Missing	638	28	666

Table S1. Demographic characteristics comparing study sample to entire NYU CHES cohort to investigate selection bias from differential loss to follow up

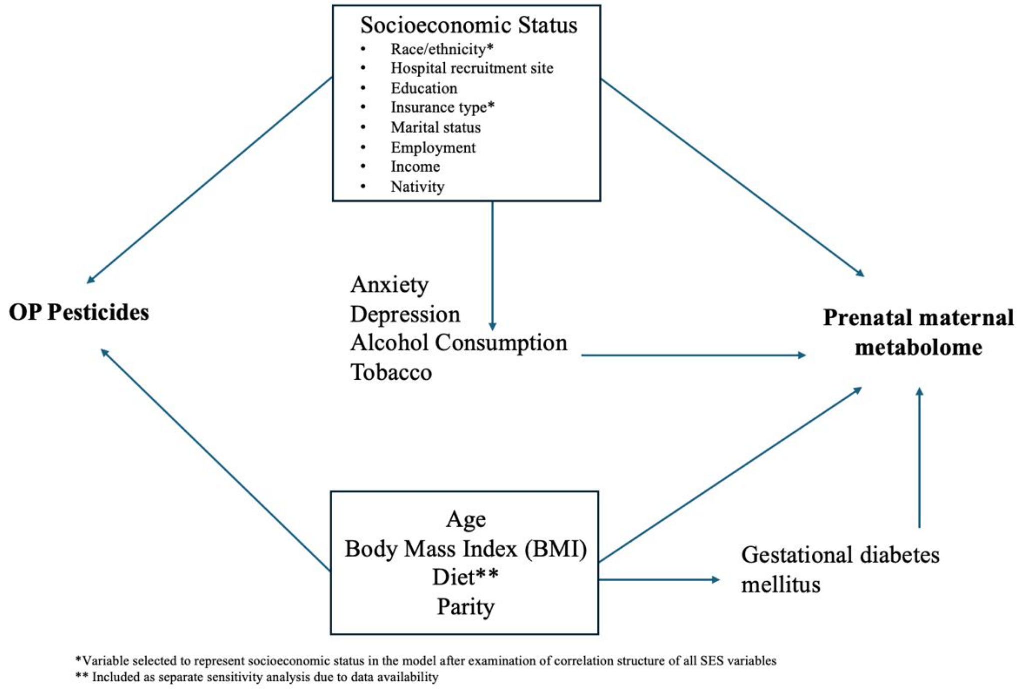

Figure S1. Directed Acyclic Graph (DAG) representing covariates relevant to OP pesticide exposure and the prenatal maternal metabolome
